# Relationship between intra-abdominal pressure and indocyanine green plasma disappearance rate: hepatic perfusion may be impaired in critically ill patients with intra-abdominal hypertension

**DOI:** 10.1186/2110-5820-2-S1-S19

**Published:** 2012-12-20

**Authors:** Manu LNG Malbrain, Dries Viaene, Andreas Kortgen, Inneke De laet, Hilde Dits, Niels Van Regenmortel, Karen Schoonheydt, Michael Bauer

**Affiliations:** 1 Department of Intensive Care, Ziekenhuis Netwerk Antwerpen (ZNA) Stuivenberg, Lange Beeldekensstraat 267, Antwerp, 2060, Belgium; 2 Department of Anaesthesiology and Intensive Care Therapy, Center for Sepsis Control and Care, Jena University Hospital, Jena, 07747, Germany

## Abstract

**Background:**

Monitoring hepatic blood flow and function might be crucial in treating critically ill patients. Intra-abdominal hypertension is associated with decreased abdominal blood flow, organ dysfunction, and increased mortality. The plasma disappearance rate (PDR) of indocyanine green (ICG) is considered to be a compound marker for hepatosplanchnic perfusion and hepatocellular membrane transport and correlates well with survival in critically ill patients. However, correlation between PDR_ICG _and intra-abdominal pressure (IAP) remains poorly understood. The aim of this retrospective study was to investigate the correlation between PDR_ICG _and classic liver laboratory parameters, IAP and abdominal perfusion pressure (APP). The secondary goal was to evaluate IAP, APP, and PDR_ICG _as prognostic factors for mortality.

**Methods:**

A total of 182 paired IAP and PDR_ICG _measurements were performed in 40 critically ill patients. The mean values per patient were used for comparison. The IAP was measured using either a balloon-tipped stomach catheter connected to an IAP monitor (Spiegelberg, Hamburg, Germany, or CiMON, Pulsion Medical Systems, Munich, Germany) or a bladder FoleyManometer (Holtech Medical, Charlottenlund, Denmark). PDR_ICG _was measured at the bedside using the LiMON device (Pulsion Medical Systems, Munich, Germany). Primary endpoint was hospital mortality.

**Results:**

There was no significant correlation between PDR_ICG _and classic liver laboratory parameters, but PDR_ICG _did correlate significantly with APP (*R *= 0.62) and was inversely correlated with IAP (*R *= -0.52). Changes in PDR_ICG _were associated with significant concomitant changes in APP (*R *= 0.73) and opposite changes in IAP (*R *= 0.61). The IAP was significantly higher (14.6 ± 4.6 vs. 11.1 ± 5.3 mmHg, *p *= 0.03), and PDR_ICG _(10 ± 8.3 vs. 15.9 ± 5.2%, *p *= 0.02) and APP (43.6 ± 9 vs. 57.9 ± 12.2 mmHg, *p* < 0.0001) were significantly lower in non-survivors.

**Conclusions:**

PDR_ICG _is positively correlated to APP and inversely correlated to IAP. Changes in APP are associated with significant concomitant changes in PDR_ICG_, while changes in IAP are associated with opposite changes in PDR_ICG_, suggesting that an increase in IAP may compromise hepatosplanchnic perfusion. Both PDR_ICG _and IAP are correlated with outcome. Measurement of PDR_ICG _may be a useful additional clinical tool to assess the negative effects of increased IAP on liver perfusion and function.

## Introduction

Intra-abdominal hypertension (IAH) and abdominal compartment syndrome (ACS) have been associated with organ dysfunction and mortality in critically ill patients [1,2]. In terms of organ dysfunction, both intra-abdominal and remote organs are involved. The effects of IAH on cardiovascular, respiratory, and renal functions have all been described in some detail [3]. Few data are available, however, on the effect of IAH on the liver [4], partly due to the fact that hepatic blood flow and liver function remain difficult to assess reliably at the bedside [5]. Liver function is routinely evaluated by plasma concentrations of liver enzymes physiologically restraint to certain cells and subcellular compartments (aspartate aminotransferase (ASAT), alanine aminotransferase (ALAT), lactate dehydrogenase (LDH), gamma-glutamyltranspeptidase (γGT), alkaline phosphatase) and laboratory parameters of liver synthesis (albumin, plasma cholinesterase, glucose, coagulation factors with international normalized ratio (INR)). However, all these tests supply only indirect information on actual liver function.

The plasma disappearance rate of indocyanine green (PDR_ICG_) might be an alternative for bedside liver function testing. After injection, indocyanine green (ICG) is distributed via the bloodstream and excreted by hepatocytes into the bile. ICG does not enter enterohepatic recirculation and is excreted completely by the gastrointestinal system. Therefore, elimination of ICG is determined by cardiac output (CO), hepatic blood flow, and hepatocellular uptake [6]. While excretion into the bile can be impaired, PDR_ICG _can be unaffected. PDR_ICG _has been shown to be a good surrogate marker for liver function and hepatosplanchnic perfusion [7-11]. Because PDR_ICG _is one of very few available markers for hepatic blood flow and function and intra-abdominal pressure (IAP) is an important indicator of a patient's physiologic status, further investigation of a possible interaction was deemed necessary because of the scarce data currently available [12-15]. Therefore, the first aim of the study was to investigate the correlation between PDR_ICG _and classic liver parameters. The second aim was to analyze the correlation between IAP, abdominal perfusion pressure (APP), and PDR_ICG _as well as between changes in IAP and APP and changes in PDR_ICG_, and finally, to determine the best threshold value for IAP, APP, and PDR_ICG _as prognostic factors in critically ill patients.

## Methods

### Patients

The study consists of a retrospective data analysis from measurements obtained in a case series of 40 critically ill patients admitted to the medical ICU of a tertiary hospital (Ziekenhuis Netwerk Antwerpen (ZNA) Stuivenberg General Hospital, Antwerp, Belgium). Disease severity on ICU admission was evaluated using the averaged simplified acute physiology score (SAPS-II), the acute physiology and chronic health evaluation (APACHE-II) score, and the sequential organ failure assessment (SOFA) score. The indication to perform PDR_ICG _was based on the clinical judgement of the attending physician in charge. The study was approved by the local institutional review board without need for informed consent due to the retrospective nature of the analysis.

### Measurements and definitions

IAP was measured using either a balloon-tipped stomach catheter connected to an IAP monitor (Spiegelberg, Hamburg, Germany, or CiMON, Pulsion Medical Systems, Munich, Germany) or a FoleyManometer (Holtech Medical, Charlottenlund, Denmark) via the bladder. IAP was measured according to the World Society of the Abdominal Compartment Syndrome (WSACS, http://www.wsacs.org) guidelines and expressed in mmHg [16]. APP was calculated as mean arterial pressure (MAP) minus IAP, and IAH was defined as a sustained or repeated pathologic elevation of IAP ≥ 12 mmHg.

The PDR_ICG _was obtained at the bedside using the LiMON device (Pulsion Medical Systems, Munich, Germany) connected to a disposable color sensor at the earlobe or finger obtaining a chromodilution curve of 0.25 mg/kg of ICG solution (in a concentration of 5 mg ICG/ml water) injected via a central venous catheter. In principle, the PDR_ICG _is determined by mono-exponential transformation of the original ICG concentration curve, backward extrapolation to the time 'zero' (100%), and describing the decay as percentage change over time. The normal range of PDR_ICG _is 18% to 25%/min in healthy subjects. Together with PDR_ICG_, the value of residual ICG after 15 min can also be calculated as a percentage.

Classic liver tests (ASAT, ALAT, LDH, γGT, alkaline phosphatase, bilirubin) and so-called liver synthesis tests (albumin, plasma cholinesterase, glucose, and coagulation factors with INR) were performed daily according to routine practice in our institution.

### Statistical analysis

Descriptive statistics are presented as mean ± standard deviation (SD) for normally distributed values and as median (with interquartile range) in case of non-normal distribution. Categorical variables were compared using the chi-squared test, while continuous variables were compared using Student's *t *test or Mann Whitney *U *test in case of non-normal distribution.

Statistical significance was defined at two-tailed *p *value levels of 0.05. Calculations were performed using SPSS software version 17 (SPSS Inc., Chicago, IL, USA). The coefficient of determination (*R*^2^) derived from Pearson's product-moment correlation (*R*) was used for measurement of correlation between the mean values of IAP, APP, and PDR_ICG _obtained in each patient. Because of repeated measurements in each patient, we used weighted analysis, as described by Bland and Altman [17,18] to investigate correlations.

To analyze whether changes in IAP or APP were related to changes or trends in PDR_ICG_, a four-quadrant trend plot was constructed by plotting ΔIAP or ΔAPP against ΔPDR_ICG _at the same time interval by subtracting the first from the last value. The concordance is calculated as the percentage of pairs with the same direction of change. Based on previous reports, the concordance should be >85% to 90%.

Receiver operating characteristics (ROC) curves were calculated (for hospital mortality), and these curves graphically describe the sensitivity of a diagnostic test (true positive proportion) vs. 1 - specificity (true negative proportion) and provide an improved measure of the overall discriminatory power of a test as they assess all possible threshold values. The WSACS recommends that a good area under the ROC (AUROC) curve is at least 0.75; the best threshold needs to be identified with a sensitivity and/or specificity of at least, or close to, 75%.

Primary endpoint was hospital mortality; secondary endpoints were ICU mortality and the development of IAH or a low PDR_ICG_. Outcome analysis and prediction was based on the best threshold identified by AUROC for APP and PDR_ICG _(lowest value) and for IAP (highest value) obtained within the first week of ICU admission for each patient.

## Results

### Correlations

**Figure 1:**
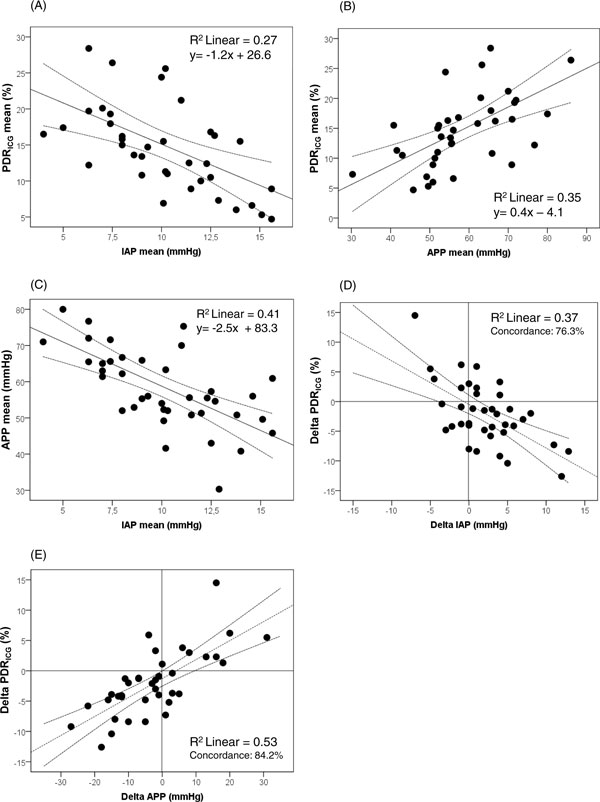
**Regression plots**. Pearson regression analysis comparing (**A**) mean intra-abdominal (IAP) and mean indocyanine green plasma disappearance rate (PDR_ICG_) per patient (*n *= 38), (**B**) mean abdominal perfusion pressure (APP) and mean PDR_ICG _per patient (*n *= 38), (**C**) mean IAP and mean APP per patient (*n *= 40) and Pearson regression analysis with four-quadrant plot comparing (**D**) mean changes in IAP (Delta IAP) with mean changes in PDR_ICG _(Delta PDR_ICG_) per patient (*n *= 38), (**E**) mean changes in APP (Delta APP) with mean changes in PDR_ICG _(Delta PDR_ICG_) per patient (*n *= 38).

The Pearson correlations between PDR_ICG _and classic liver laboratory parameters, general hemodynamic parameters (MAP) and lactate, were not significant. A total of 182 paired IAP, APP, and PDR_ICG _measurements were performed in 40 patients; however, in two patients, only one PDR_ICG _value was obtained. Analysis according to Pearson is shown in Figure 1 and revealed that the PDR_ICG _significantly correlated with APP (*R *= 0.62, *p* < 0.0001) (panel A) and inversely correlated with IAP (*R *= -0.52, *p *= 0.001) (panel B); not surprisingly, the higher the IAP, the lower is the APP (*R *= -0.64, *p* < 0.0001) (panel C). Furthermore, changes in IAP (*n *= 38, since only one paired measurement was obtained in two patients) were associated with significant but opposite changes in PDR_ICG _with 76.3% concordance (*R *= -0.61, *p* < 0.0001) (panel D), while changes in APP (*n *= 38) were associated with significant concomitant changes in PDR_ICG _with 84.2% concordance (*R *= 0.73, *p* < 0.0001) (panel E). These correlations were, however, only weak and did not reach the *R*^2 ^> 0.6 threshold.

### Outcome

**Figure 2:**
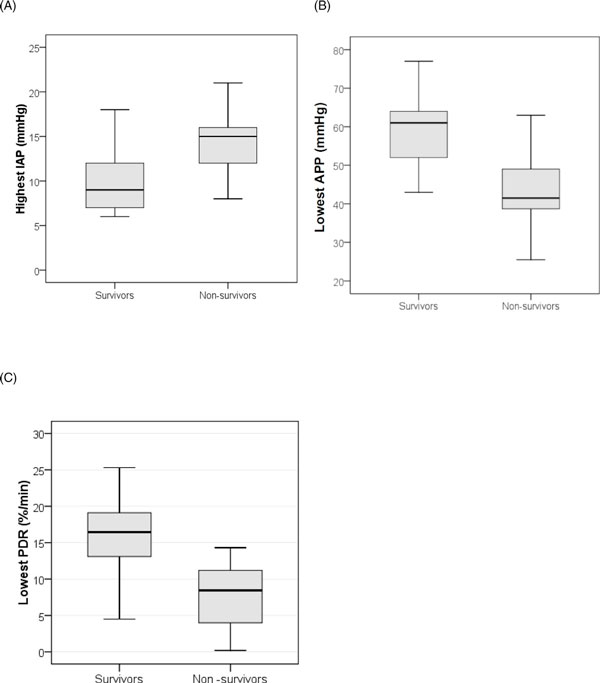
**Box plots comparing survivors with non-survivors**. (**A**) Highest IAP values. (**B**) Lowest APP values. (**C**) Lowest PDR_ICG _values.

Comparing hospital survivors (*n *= 14) with non-survivors (*n *= 26), we observed significantly higher values of IAP (14.6 ± 4.6 vs. 11.1 ± 5.3 mmHg, *p *= 0.03) and significantly lower values of APP (43.6 ± 9 vs. 57.9 ± 12.2 mmHg, *p* < 0.001) and PDR_ICG _(10 ± 8.3 vs. 15.9 ± 5.2%, *p *= 0.02) in non-survivors (Figure 2). Table 1 shows the baseline patient characteristics according to outcome (hospital mortality). Table 2 shows the worst values for IAP, APP, and PDR_ICG _in non-survivors. The incidence of IAH (defined as a mean IAP ≥ 12 mmHg) and low PDR_ICG _(defined as <12%) was significantly higher in non-survivors (Table 2).

**Table 1:** Patient demographics and baseline characteristics in survivors and non-survivors

	Total	Survivors	Non-survivors	*p *value
Patients, *n *(%)	40	14 (35)	26 (65)	
Age (years)	59.8 ± 14.1	54.8 ± 16.1	62.4 ± 12.4	NS
Body mass index (kg/m^2^)	25.4 ± 6.3	27.7 ± 8.6	24.2 ± 4.4	NS
Gender (M/F)	1:1	1:1	1:1	NS
ICU stay (days)	14.5 (8.3 to 23.8)	10.5 (6.8 to 34.3)	16 (10 to 22.3)	NS
Hospital stay (days)	27 (13.5 to 58.5)	51.5 (18.3 to 85)	20 (12.8 to 32.3)	0.059
SAPS II	45.3 ± 15.8	33.9 ± 9	51.4 ± 15.3	<0.0001
SAPS death probability (%)	43.1 ± 26.3	21.4 ± 15.7	54.7 ± 23.4	<0.0003
APACHE II	24 ± 14.2	18.3 ± 11.9	27 ± 14.6	0.06
SOFA	9 ± 3.5	6.6 ± 2.9	10.3 ± 3.1	0.001
SOFA respiratory	2.2 ± 1.1	1.6 ± 1.2	2.5 ± 0.9	0.02
SOFA coagulation	1.1 ± 1.3	0.8 ± 1.3	1.2 ± 1.4	NS
SOFA liver	1 ± 1.2	0.9 ± 1.3	1 ± 1.1	NS
SOFA cardiovascular	2.3 ± 1.6	1.3 ± 1.4	2.8 ± 1.4	0.002
SOFA neurological	0.8 ± 1.2	0.4 ± 0.9	1.1 ± 1.3	0.07
SOFA renal	1.4 ± 1.4	1.4 ± 1.5	1.4 ± 1.4	NS
MAP admission (mmHg)	70.9 ± 12.5	78.8 ± 13.6	66.7 ± 9.7	0.002
IAP admission (mmHg)	8.9 ± 3.9	7.8 ± 4.1	9.5 ± 3.7	NS
APP admission (mmHg)	59 ± 13.9	67.1 ± 16.1	54.7 ± 10.6	0.006
PDR admission (%)	16.4 ± 6.6	18 ± 5.1	15.6 ± 7.3	NS
Fluid balance admission (ml)	1,215 (−166 to 3,178)	592 (−656 to 2,705)	1,591 (−67 to 3,546)	NS

**Table 2:** Outcome predictors in survivors and non-survivors

	Total	Survivors (*n *= 14)	Non-survivors (*n *= 26)	*p *value
Highest IAP (mmHg)	13.4 ± 5.1	11.1 ± 5.3	14.6 ± 4.6	0.03
Day highest IAP	4 (2 to 6.8)	3 (1 to 5.3)	5 (2.8 to 7)	NS
Lowest APP (mmHg)	48.6 ± 12.2	57.9 ± 12.2	43.6 ± 9	<0.0002
Day lowest APP	4 (1.3 to 7)	2 (1 to 3)	5 (2.8 to 7)	0.007
Lowest PDR (%)	12.1 ± 7.8	15.9 ± 5.2	10 ± 8.3	0.02
Day lowest PDR	5 (2 to 7)	2 (1 to 5)	5.5 (3.8 to 7)	0.03
IAH, *n *(%)	25 (62.5)	4 (28.6)	21 (80.8)	0.002
PDR_ICG _< 12%, *n *(%)	23 (57.5)	3 (21.4)	20 (76.9)	0.001

### Determination of thresholds

**Figure 3:**
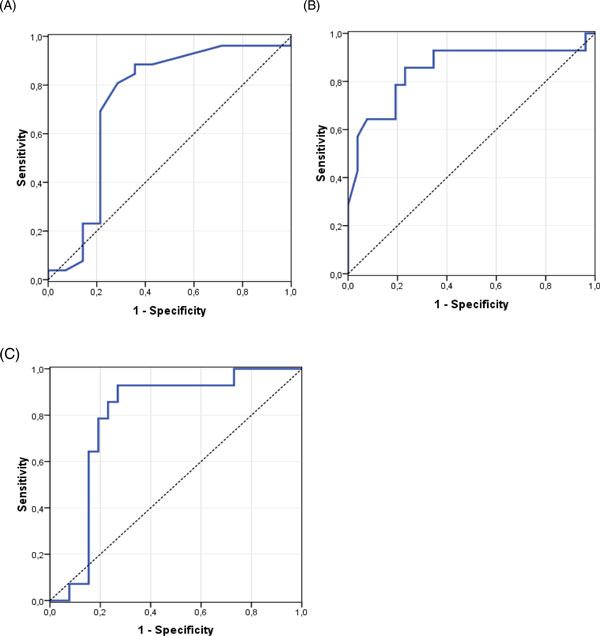
**ROC curves**. (**A**) Highest IAP. (**B**) Lowest APP. (**C**) Lowest PDR_ICG._

Analysis with ROC curves showed a significant AUROC curve of 0.84, 0.74, and 0.82 respectively for SAPS-II, APACHE-II, and SOFA scores. ROC curve analysis for the highest IAP showed an AUROC of 0.74 (95% confidence interval (CI), 0.55 to 0.93; *p *= 0.014). An IAP < 12mmHg had a sensitivity of 80.8% and a specificity of 71.4% for good outcome (Figure 3, panel A). ROC curve analysis for the lowest APP showed the best results with an AUROC of 0.85 (95% CI, 0.71 to 0.99; *p* < 0.0001). An APP ≥ 52.5 mmHg had a sensitivity of 71.4% and a specificity of 80.8% for good outcome (Figure 3, panel B), and finally, ROC curve analysis for the lowest PDR showed an AUROC of 0.79 (95% CI, 0.64 to 0.94; *p *= 0.003). A PDR ≥ 12% had a sensitivity of 78.6% and a specificity of 80.8% for good outcome (Figure 3, panel C).

Univariate analysis comparing baseline characteristics according to IAP and PDR_ICG _thresholds is shown in Tables 3 and 4. Patients with IAH and a PDR_ICG _< 12% had significantly higher ICU and hospital mortality, and the incidence of IAH was higher in patients with low PDR_ICG _and vice versa (Tables 5 and 6).

**Table 3:** Demographics and baseline characteristics in patients with normal and low PDR_ICG_

	Lowest PDR ≥ 12	Lowest PDR < 12	*p *value
Patients, *n *(%)	17 (42.5)	23 (57.5)	
Age (years)	59.6 ± 15.7	59.9 ± 13.1	NS
Body mass index (kg/m^2^)	26.4 ± 8.6	24.7 ± 4	NS
SAPS II	43.7 ± 14.9	46.4 ± 16.6	NS
SAPS death probability (%)	38.1 ± 27.4	46.7 ± 25.4	NS
APACHE II	21.9 ± 11.2	25.5 ± 16.1	NS
SOFA	7.9 ± 3.9	9.8 ± 3.1	NS
MAP admission (mmHg)	73.1 ± 15	69.3 ± 10.4	NS
IAP admission (mmHg)	7.5 ± 2.7	9.9 ± 4.3	0.05
APP admission (mmHg)	63 ± 15	56.1 ± 12.7	NS
PDR admission (%)	21.2 ± 5.9	12.9 ± 4.7	<0.0001

**Table 4:** Demographics and baseline characteristics in patients without and with IAH

	No IAH	IAH	*p *value
Patients, *n *(%)	15 (37.5)	25 (62.5)	
Age (years)	56.8 ± 16.8	61.5 ± 12.2	NS
Body mass index (kg/m^2^)	24.3 ± 4.1	26.1 ± 7.4	NS
SAPS II	39.5 ± 14	48.8 ± 16	0.07
SAPS death probability (%)	33.2 ± 28.5	49 ± 23.4	0.06
APACHE II	21.2 ± 16.5	25.6 ± 12.7	NS
SOFA	6.7 ± 3.1	10.4 ± 3	0.001
MAP admission (mmHg)	76.5 ± 14.1	67.6 ± 10.4	0.03
IAP admission (mmHg)	6.3 ± 2.1	10.5 ± 3.9	<0.0001
APP admission (mmHg)	68.2 ± 13.7	53.6 ± 11.1	0.001
PDR admission (%)	18.7 ± 6.8	15 ± 6.3	0.09

**Table 5:** Study endpoints in patients with normal and low PDR_ICG_

	Lowest PDR ≥ 12 (*n *= 17)	Lowest PDR < 12 (*n *= 23)	*p *value
Highest IAP (mmHg)	11 ± 4.5	15.1 ± 4.8	0.01
Lowest APP (mmHg)	55.6 ± 12.1	43.5 ± 9.6	0.001
Lowest PDR (%)	19.4 ± 5.8	6.6 ± 3.3	<0.0001
IAH, *n *(%)	6 (35.3)	19 (82.6)	0.003
ICU mortality, *n *(%)	4 (23.5)	18 (78.3)	0.001
Hospital mortality, *n *(%)	6 (35.3)	20 (87)	0.001

**Table 6:** Study endpoints in patients without and with IAH

	No IAH (*n *= 15)	IAH (*n *= 25)	*p *value
Highest IAP (mmHg)	8.3 ± 1.9	16.4 ± 3.7	0.000
Lowest APP (mmHg)	59.9 ± 9.5	41.9 ± 8	<0.0001
Lowest PDR (%)	17.1 ± 7.1	9.1 ± 6.7	0.001
PDR_ICG _< 12%, ***n ***(%)	4 (26.7)	19 (76)	0.003
ICU mortality, *n *(%)	5 (33.3)	17 (68)	0.04
Hospital mortality, *n *(%)	5 (33.3)	21 (84)	0.002

### Evolution of main study parameters in relation to outcome

**Figure 4:**
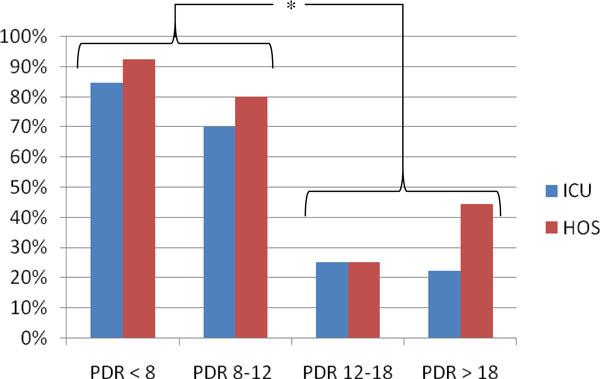
**ICU and hospital mortality according to PDR_ICG _category**. The asterisk indicates a *p *value of 0.006 comparing ICU and hospital (HOS) outcome in the group of patients with a PDR_ICG _below and higher than 12%.

The lower the PDR_ICG_, the higher are the ICU and hospital mortality (Figure 4). Analysis with contingency tables and the Fisher exact test showed significant differences in outcome comparing patients with PDR_ICG _below or higher than 12% with a *p *value of 0.006 for both ICU and hospital mortality.

**Figure 5:**
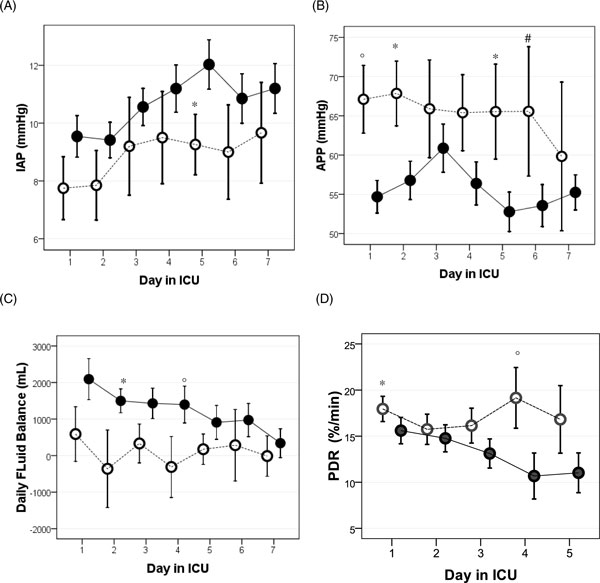
**Day-by-day evolution (presented as a plot of means with SD error bars) of important parameters**. This is during the first week of ICU stay stratified between survivors (open circles) and non-survivors (closed circles). (**A**) Evolution of IAP; the asterisk indicates a *p *value of 0.05. (**B**) Evolution of APP; the asterisk indicates a *p *value of 0.03, the degree sign indicates a *p *value of 0.06, and the number sign indicates a *p *value of 0.08. (**C**) Evolution of daily fluid balance; the asterisk indicates a *p *value of 0.03 and the degree sign indicates a *p *value of 0.08. (**D**) Evolution of PDR_ICG_; the asterisk indicates a *p *value of 0.06 and the degree sign indicates a *p *value of 0.05.

During the first week of ICU stay, survivors had higher APP and PDR_ICG_, lower IAP, and a less positive daily fluid balance; in fact, survivors had a negative to zero fluid balance from day 2 (Figure 5). Non-survivors developed their worst values for IAP, APP, and PDR_ICG _later on during the ICU stay (Table 2 and Figure 5).

## Discussion

The data of this retrospective analysis suggest that IAP is inversely correlated with PDR_ICG_. The rationale behind this observation could be that increased IAP leading to decreased APP and a drop in CO can result in compromised splanchnic and thereby hepatic perfusion. As PDR_ICG _is dependent from both hepatocellular function and effective sinusoidal perfusion, this consecutively leads to reduced PDR_ICG_. This is underlined in our study by the good correlation between changes in IAP (and APP) with changes in PDR_ICG_. Rapid changes (within hours) are most likely due to changes in sinusoidal perfusion rather than hepatocellular uptake of the dye. Measuring PDR_ICG _might therefore be a good additional tool to estimate influence of IAH on splanchnic perfusion in the individual patient.

There are very few data on the relationship between IAP and PDR_ICG_. So far, Michelet et al. studied this subject prospectively in detail [13]. In 20 ARDS patients, they looked at the influence of prone positioning on IAP, PDR_ICG_, and extravascular lung water compared to the supine position, lying on a conventional foam mattress vs. an air-cushioned mattress. They observed an increase in IAP and a decrease in PDR_ICG _in the prone compared with the supine position on a conventional foam mattress. The effect of proning on IAP was the topic of a recent review [19]. The use of an air-cushioned mattress had beneficial effects on IAP and PDR_ICG _during the prone position. Analyzing our data, we found that PDR_ICG _correlated significantly with IAP and even more so with APP. Similar to the data of Michelet et al. [13], changes in IAP were associated with significant concomitant but opposite changes in PDR_ICG_, suggesting that an increase in IAP may compromise hepatosplanchnic perfusion. Hering et al., however, did not observe a decrease in PDR_ICG _when patients were proned despite the small increase in IAP from 10 to 13 mmHg [15].

Previous studies have shown that overall mortality increases with lower values of PDR_ICG _and that a PDR_ICG _threshold >14% is a good predictor of survival [20,21]. With respect to survival and using analysis by ROC with AUROC curve as a measure of accuracy, Inal et al. [10] demonstrated a superior sensitivity and specificity (AUROC = 0.78) of PDR_ICG _compared to the APACHE-II score (AUROC = 0.64), SOFA score (AUROC = 0.56), and bilirubin (AUROC = 0.62), while Sakka and Meier-Hellmann [20] showed a superior sensitivity and specificity to APACHE-II (AUROC = 0.68) and a comparable sensitivity and specificity to SAPS-II (AUROC = 0.76). In a prospective observational study in septic patients, PDR_ICG _< 8% predicted mortality with high sensitivity and specificity [22]. This was confirmed by our data, showing significantly lower values of PDR_ICG _in non-survivors. We also noted significantly higher IAP and lower APP values in non-survivors, suggesting that low IAP and high APP may be useful predictors of survival in ICU patients and thus may also be interesting resuscitation endpoints, especially, since we observed that changes in IAP and APP were related to changes in PDR_ICG_. In non-survivors, the worst values of IAP, APP, and PDR_ICG _occurred later on during the course of the critical illness, suggesting that non-resolution of IAH and sustained poor hepatosplanchnic perfusion eventually may lead to organ dysfunction and increased mortality. More recently, Inal et al. showed in a retrospective analysis of 30 critically ill patients that IAP was significantly higher (21.5 ± 2 mmHg vs. 11.7 ± 1.5 mmHg) and PDR_ICG _was significantly lower (10.9 ± 3.4% vs. 24.5 ± 6.8%) in non-survivors compared to survivors [10].

Our data show no significant correlation between PDR_ICG _and conventional liver laboratory tests in mixed ICU patients. Two possible explanations for this observation might be that either PDR_ICG _does not reflect liver function at all or PDR_ICG _gives additional information on hepatocellular function and hepatosplanchnic perfusion that is not 'detected' by the classic liver function tests. However, due to its unique hepatic elimination, PDR_ICG _has been shown to be a good surrogate marker for liver function and hepatosplanchnic perfusion; therefore, the latter explanation seems more likely [10,11,20].

Our study has several limitations. First, the data sample is quite small with only 40 patients studied. Second, the analysis was retrospective, so we could not examine the effects of interventions to lower IAP or to improve APP on values of PDR_ICG_. Third, our results are merely observational, so we cannot exclude other confounding factors and deduct cause and effect from the observed relations. Finally, important data on CO are missing, and this is an important parameter in order to understand and interpret the possible effects of IAP on liver flow (and thus also on PDR_ICG_), since previous studies showed that liver flow correlates well with CO.

## Conclusions

In this study, we demonstrated that PDR_ICG _is not correlated with classic liver laboratory tests; hence, it may provide additional information on hepatic blood flow and hepatocellular function. We found a significant correlation between PDR_ICG _and IAP (and APP), suggesting that IAH may impair hepatic blood flow and/or hepatic function as it does also compromise other organ functions. Finally, we found that there were significant differences between survivors and non-survivors regarding IAP, APP, and PDR_ICG_. An IAP below 12 mmHg, a PDR_ICG _above 12%/min, and an APP above 52.5 mmHg were predictive for good outcome. PDR_ICG _might be a good tool to estimate clinical impact of IAH on splanchnic perfusion in selected critically ill patients.

## Abbreviations

ALAT: alanine aminotransferase; APACHE: acute physiology and chronic health evaluation; APP: abdominal perfusion pressure; ASAT: aspartate aminotransferase; AUROC: area under the ROC curve; γGT: gamma-glutamyltranspeptidase; IAH: intra-abdominal hypertension; IAP: intra-abdominal pressure; ICG: indocyanine green; INR: international normalized ratio; LDH: lactate dehydrogenase; MAP: mean arterial pressure; PDR: plasma disappearance rate; PDR_ICG_: plasma disappearance rate of indocyanine green; *R*: Pearson regression coefficient; ROC: receiver operating characteristics; SAPS: simplified acute physiology score; SOFA: sequential organ failure assessment.

## Competing interests

MB and MLNGM are members of the medical advisory board of Pulsion Medical Systems (Munich, Germany), a monitoring company. The other authors declare that they have no competing interests. AK received a study grant from Pulsion Medical Systems.

## Authors' contributions

DV, IDL, NVR, KS, HD, and MLNGM planned the study and were responsible for the design, coordination, and drafting the manuscript. AK and MB participated in the study design and helped draft the manuscript. DV and MLNGM performed the statistical analysis and helped draft the manuscript. All authors read and approved the final manuscript.
